# Editorial: Clinical translation and commercialisation of advanced therapy medicinal products, volume II

**DOI:** 10.3389/fbioe.2026.1746333

**Published:** 2026-01-27

**Authors:** Yves Bayon, Alain A. Vertès

**Affiliations:** 1 Medtronic-Sofradim Production, Trévoux, France; 2 NxR Biotechnologies GmbH, Basel, Switzerland

**Keywords:** advanced therapy medicinal products (ATMPs), clinical translation, drug repurposing, foreign body response (FBR), interventional procedure, regulatory challenges, scalable manufacturing

## Introduction

The landscape of medical innovation is being fundamentally transformed by bioengineering, that is, —the application of engineering principles to design and manipulate biological systems. The past decade has witnessed the commercial validation of Advanced Therapy Medicinal Products (ATMPs)—gene, cell, and tissue-engineered products, typically of human origin—shifting these treatments from scientific promise to clinical reality. Yet, the total impact of bioengineering is far broader. Today, the field is undergoing a dual revolution, simultaneously driving the next generation of ATMPs and strengthening a diverse pipeline of commercially viable, non-ATMP therapeutic solutions.

This revolution is fueled by a transition from proving clinical feasibility to overcoming the phenomenon of “valley of death” experienced by many biotech companies, through scalable manufacturing and regulatory alignment. As ATMPs expand from ultra-rare diseases to broader indications, they are redefining standard-of-care paradigms. Parallel to this, bioengineering is delivering high-fidelity non-ATMP interventions that prioritize manufacturability and interventional precision.

## Key achievements and next moves of ATMPs

A 2018 perspective on the field was published in this journal’s Research Topic, *Clinical Translation and Commercialisation of Advanced Therapy Medicinal Products*, charted the early success of ATMPs (Yu et al.). That paper highlighted landmark approvals such as the first gene and CAR-T cell therapies, validating the transformative potential of genetic medicine. It also cautioned that despite these breakthroughs, the first generation of therapies was hampered by numerous challenges comprising: lengthy development timelines, complex and high-cost manufacturing, and expensive treatments associated with persistent reimbursement hurdles.

Fast-forwarding to today, the challenge has shifted from “Can we cure it?” to “Can we scale the cure?” The newest generation of ATMPs, powered by advanced bioengineering, is directly addressing those limitations (Sanchez-Guijo et al., 2024; Dahiya et al., 2025).

## Focused summary on next moves: scaling and efficacy driven by innovative technologies

The future of advanced therapies is focused on overcoming the logistical, cost, and safety limitations of the first generation, notably by leveraging new technologies as key enablers, as illustrated by striking examples below.

The most impactful next move in cell therapy is the shift from autologous to allogeneic (that is. donor-derived) “off-the-Shelf” CAR-T cell products. Allogeneic products are aimed at becoming more broadly and instantly available, cheaper treatment, bypassing the weeks long manufacturing constraints of patient-specific therapies. For most cell types, this is only possible through precise gene editing (enabled by the technologies CRISPR and TALENs) to modify donor cells such as to prevent them from causing potentially life-threatening Graft-versus-Host Disease (GvHD). On the other hand, mesenchymal stem cells that have immunosuppressive properties have been demonstrated to be immune-evasive; as a result they can be used allogeneically to treat a variety of conditions, with the first such product having been approved by the FDA in December 2024 to treat steroid-refractory acute paediatric GvHD.

Another key achievement is provided by *in vivo* gene editing (Macarrón Palacios et al., 2024; Volodina and Smirnikhina, 2025). Instead of modifying cells outside the body, this approach delivers the gene-editing machinery directly to the patient’s affected tissue, often using non-viral carriers like Lipid Nanoparticles (LNPs). This aims to convert complex procedures into a non-surgical, single-injection therapeutic model, opening the door to treat common genetic diseases at scale.

To move beyond blood cancers to address solid tumors, “Armored CARs” have been bioengineered (Pievani et al., 2024; Yang et al., 2025). These are T-cells genetically modified to secrete therapeutic molecules (like IL-12) or to resist the harsh, immune-suppressive environment of solid tumors, finally giving cell therapy a foothold in the most common cancer types.

## Maturation of the ATMP market: from first approvals to commercial expansion

The maturation of the ATMP market has transitioned into a commercially sophisticated phase, moving beyond initial breakthroughs to a diverse portfolio of therapies that offer curative potential for conditions where conventional treatments have failed. This evolution is characterized by a shift from proving clinical feasibility to achieving scalable impact across three primary classes, with several landmark approvals occurring since 2018 (see the text below and Table 1 for a summary).

**TABLE 1:** Summary of striking ATMP case studies (Post-2018 approvals).

Therapy class	Striking example	Key technology	FDA/EMA approval	Estimated cost per patient (single dose/Total therapy)
GTMP	Hemgenix	AAV vector (factor IX)	2022/2023	$3.5 million
sCTMP	Carvykti	Autologous CAR-T	2022/2022	∼$465 k–$593 k
Precision/RNA	Casgevy	CRISPR-Cas9	2023/2024	$2.2 million
Precision/RNA	Leqvio	siRNA	2021/2020	∼$7,000/year

The GTMP market is currently dominated by AAV-vector technologies designed to replace or repair defective genes with a single, transformative dose.

- Zolgensma (Pharmaphorum, 2025) serves as a striking example for Spinal Muscular Atrophy, offering a “one-and-done” solution for a rare condition affecting 1 in 10,000 births, with a wholesale cost of approximately $2.1 to $2.25 million.
- Hemgenix (GeneOnline News, 2025) represents a similar leap for Hemophilia B, providing long-term production of essential clotting factors at a cost of $3.5 million per patient.

The sCTMP class utilizes living cells—often the patient’s own—to fight disease, particularly in oncology and complex chronic conditions.

- Breyanzi (CURE, 2024) and Carvykti price (2025) are powerful autologous CAR-T cell therapies for blood cancers like Large B-cell Lymphoma and Multiple Myeloma, with treatment costs ranging from roughly $410,000 to nearly $600,000.
- Alofisel (Omedit, 2025) demonstrates the expansion into allogeneic (donor-derived) mesenchymal stem cell therapy, offering a targeted treatment for complex Crohn’s Perianal Fistulas.

Precision Genetic and RNA-Based Therapies is an emerging category of products that includes both permanent gene editing and temporary gene-silencing mechanisms.

- Casgevy (Pagliarulo, 2023) made history as the first approved CRISPR-Cas9 therapy, providing a functional cure for Sickle Cell Disease and Beta-Thalassemia for approximately $2.2 million.
- Onpattro (Sehgal et al., 2024) and Leqvio (Drugs.com, 2025) utilize siRNA (RNA interference); while Onpattro addresses an ultra-rare amyloidosis at over $375,000 per year, Leqvio represents the shift toward common diseases, treating Hypercholesterolemia for roughly $7,000 per year.

## Diversification: bioengineering beyond ATMPs

Critically, the bioengineering revolution is not solely governed by ATMPs. The field has matured by focusing on mechanistic, manufacturable, and commercially savvy interventions that address specific clinical gaps, often leveraging existing approved components.

From the articles of the Research Topic *Clinical Translation and Commercialisation of Advanced Therapy Medicinal Products, Volume II of Frontiers in Bioengineering and Biotechnology*, a clear diversification is observed across three major themes reflecting current trends to generate added clinical value. Drug repurposing), or repositioning, a first major theme, is a strategic approach that finds new therapeutic applications for existing drugs—those already approved, withdrawn, or in advanced development—outside their initial intended use (Xia et al., 2024; Pinzi et al., 2024). This method generates significant added clinical value by leveraging established safety and toxicity profiles, which dramatically reduces the high costs, lengthy timelines, and substantial attrition rates associated with traditional *de novo* drug discovery. Consequently, drug repurposing can accelerate drug development, potentially bringing treatments to market in a fraction of the time and cost, and offers a crucial pathway for addressing unmet medical needs. Surgical technique is crucial to the clinical success of biomaterials (Kalashnikov et al., 2025). Proper implantation, minimal tissue damage, and good sterility reduce inflammation and implant failure. Optimizing the surgical procedure is therefore essential to ensure or improve long-term outcomes. Improving biomaterials is another way to directly enhance clinical value (Zhang et al., 2022). Better biocompatibility and safety reduce complications and costs. Controlled degradation and mechanical matching improve tissue integration and long-term function. Targeted delivery increases treatment effectiveness with fewer side effects. Finally, better manufacturability and solid clinical evidence make approval and adoption more likely.

These three dimensions of bioengineering will be illustrated by the three articles in this Research Topic.

1. Repurposed Drugs via Novel Delivery Routes: This involves using an approved drug but developing a new delivery system to reach an inaccessible target. A remarkable example from this Research Topic is the study by Yoshida et al., which addressed Congenital Diaphragmatic Hernia (CDH) by delivering Sildenafil—a drug originally for erectile dysfunction—directly into the amniotic fluid. This intra-amniotic (IA) delivery allows the fetus to “breathe” and swallow the medication, facilitating direct lung uptake that significantly improves pulmonary blood flow and vascular health without risking maternal exposure. Such research perfectly aligns with the Research Topic’s focus on Clinical Translation & Commercialisation, as it demonstrates how innovative delivery engineering can bypass traditional pharmacological hurdles to provide scalable, high-fidelity prenatal interventions for life-threatening developmental disorders.
2. Integrated Surgical Devices with Clear Interventional Pathways: Device-based bioengineering moves from complex, concept-only scaffolds to practical, scalable clinical tools. In a significant contribution to the Research Topic, Ying et al. addressed the high recurrence rate of Lumbar Disc Herniation (LDH)—which can reach up to 15% post-discectomy—by developing a novel suturing-guide annulus closure device (ACD). Recognizing that large defects in the poorly regenerative and low-vascularity annulus fibrosus (AF) often lead to reherniation, and that existing solutions are frequently expensive or unsuitable for endoscopic use, the team engineered a simple, stainless-steel suture guide designed specifically for microendoscopic application. This bioengineering solution allows surgeons to precisely puncture AF edges and secure them with non-absorbable sutures to effectively “close” the defect; in *ex-vivo* sheep models, the device demonstrated a significantly higher mechanical failure load compared to both non-sutured controls and traditional hand-stitching due to its ability to achieve deeper needle penetration and a more robust seal. This work nicely showcased how a practical, scalable, and cost-effective interventional pathway can bridge the gap between complex experimental scaffolds and manufacturable clinical tools ready for commercial adoption.
3. Biomaterials Optimized for Integration and Scalability (Yang et al.): The focus shifts from simply “biocompatible” materials to those that actively promote function and safety. In a significant contribution to the Research Topic, Yang et al. addressed the long-term failure of synthetic vascular grafts—often caused by thrombosis and poor healing—by developing uncrosslinked porcine collagen-coated vascular grafts (UPCCVG). By avoiding traditional chemical crosslinking, this innovation preserves the natural triple-helix structure of collagen to provide a “native-like” scaffold that host cells readily recognize. In porcine models, these grafts demonstrated excellent patency and promoted functional endothelialization, creating a healthy inner lining that naturally prevents clot formation. This work perfectly illustrated the upgrading of existing, commercially validated platforms with an advanced, low-immunogenicity coating that avoids complex regulatory hurdles. Ultimately, the UPCCVG offers a faster, more scalable pathway to market, closing the loop between scientific innovation and practical clinical utility to reduce the burden of vascular re-interventions.

## Final thoughts: from innovation to scalable clinical impact

Bioengineering now stands at the intersection of scientific innovation and clinical translation. Its dual revolution is shaping both the next-generation of Advanced Therapy Medicinal Products (ATMPs) and a growing ecosystem of practical, scalable solutions—from repurposed drugs and surgical devices to optimized biomaterials. Achieving true clinical impact requires early and sustained commitment from all stakeholders in product development. For innovators, the key to success is synchronization: pairing robust science with scalable GMP manufacturing, securing early regulatory engagement (whether for a device or a drug), and establishing health-economics modeling to ensure reimbursement and market adoption. The future of medicine lies in this comprehensive, engineered approach.
